# Adjuvant holmium-166 radioembolization after radiofrequency ablation in early-stage hepatocellular carcinoma patients: a dose-finding study (HORA EST HCC trial)

**DOI:** 10.1007/s00259-024-06630-z

**Published:** 2024-02-08

**Authors:** Pim Hendriks, Daphne D. D. Rietbergen, Arian R. van Erkel, Minneke J. Coenraad, Mark J. Arntz, Roel J. Bennink, Andries E. Braat, Stijn Crobach, Otto M. van Delden, Petra Dibbets-Schneider, Tom van der Hulle, Heinz-Josef Klümpen, Rutger W. van der Meer, J. Frank W. Nijsen, Catharina S. P. van Rijswijk, Joey Roosen, Bastian N. Ruijter, Frits Smit, Mette K. Stam, R. Bart Takkenberg, Maarten E. Tushuizen, Floris H. P. van Velden, Lioe-Fee de Geus-Oei, Mark C. Burgmans, Dutch Hepatocellular and Cholangiocarcinoma Group

**Affiliations:** 1 Interventional Radiology Research (IR2) Group, Department of Radiology, Leiden University Medical Center, P.O. Box 9600, 2300 RC Leiden, The Netherlands; 2 Section of Nuclear Medicine, Department of Radiology, Leiden University Medical Center, Leiden, The Netherlands; 3 Department of Gastroenterology and Hepatology, Leiden University Medical Center, Leiden, The Netherlands; 4 Department of Medical Imaging, Radboud University Medical Center, Nijmegen, The Netherlands; 5 Department of Radiology and Nuclear Medicine, Amsterdam University Medical Centers, Amsterdam, The Netherlands; 6 Department of Surgery, Leiden University Medical Center, Leiden, The Netherlands; 7 Department of Pathology, Leiden University Medical Center, Leiden, The Netherlands; 8 Department of Medical Oncology, Leiden University Medical Center, Leiden, The Netherlands; 9 Department of Medical Oncology, Amsterdam University Medical Centers, Amsterdam, The Netherlands; 10 Department of Gastroenterology and Hepatology, Amsterdam University Medical Centers, Amsterdam, The Netherlands; 11 Biomedical Photonic Imaging Group, TechMed Center, University of Twente, Enschede, The Netherlands; 12 Department of Radiation Sciences & Technology, Delft University of Technology, Delft, The Netherlands

**Keywords:** Hepatocellular carcinoma, Radiofrequency ablation, Trans-arterial radioembolization, Holmium-166, Adjuvant therapy, Dose-escalation study

## Abstract

**Purpose:**

The aim of this study was to investigate the biodistribution of (super-)selective trans-arterial radioembolization (TARE) with holmium-166 microspheres (^166^Ho-MS), when administered as adjuvant therapy after RFA of HCC 2–5 cm. The objective was to establish a treatment volume absorbed dose that results in an absorbed dose of ≥ 120 Gy on the hyperemic zone around the ablation necrosis (i.e., target volume).

**Methods:**

In this multicenter, prospective dose-escalation study in BCLC early stage HCC patients with lesions 2–5 cm, RFA was followed by (super-)selective infusion of ^166^Ho-MS on day 5–10 after RFA. Dose distribution within the treatment volume was based on SPECT-CT. Cohorts of up to 10 patients were treated with an incremental dose (60 Gy, 90 Gy, 120 Gy) of ^166^Ho-MS to the treatment volume. The primary endpoint was to obtain a target volume dose of ≥ 120 Gy in 9/10 patients within a cohort.

**Results:**

Twelve patients were treated (male 10; median age, 66.5 years (IQR, [64.3–71.7])) with a median tumor diameter of 2.7 cm (IQR, [2.1–4.0]). At a treatment volume absorbed dose of 90 Gy, the primary endpoint was met with a median absorbed target volume dose of 138 Gy (IQR, [127–145]). No local recurrences were found within 1-year follow-up.

**Conclusion:**

Adjuvant (super-)selective infusion of ^166^Ho-MS after RFA for the treatment of HCC can be administered safely at a dose of 90 Gy to the treatment volume while reaching a dose of ≥ 120 Gy to the target volume and may be a favorable adjuvant therapy for HCC lesions 2–5 cm.

**Trial registration:**

Clinicaltrials.gov NCT03437382. (registered: 19-02-2018)

## Introduction

In the management of hepatocellular carcinoma (HCC), thermal ablation (TA) has become the preferred curative treatment for lesions up to 2 cm, owing to its equal effectiveness and lower complication rate compared to surgical techniques [1, 2]. For larger tumors, surgical resection is generally regarded as the recommended treatment, provided that liver function is preserved [1–7]. Nevertheless, most patients are not eligible for surgery due to the presence of underlying liver cirrhosis induced portal hypertension, impaired liver function, other comorbidity, and/or an unfavorable tumor location [1]. As a result, these patients are often treated with TA or trans-arterial therapies, such as trans-arterial chemoembolization (TACE) or trans-arterial radioembolization (TARE) [1, 2].

The risk of developing local recurrence after TA is generally considered to be higher than after surgical resection, especially for lesions > 3 cm [5, 6, 8]. Local recurrences are mainly caused by (a) insufficient heat propagation during thermal ablation, (b) heat sink effect in case of tumors with a bordering intrahepatic vessel, or (c) the presence of viable satellite nodules. Most recurrences are found in the periphery of, or in close proximity to the treated tumor [9].

In order to reduce local recurrence rates after TA of larger lesions (> 3 cm), the combined treatment of TA with TACE has been studied previously. Although the combined treatment may improve survival as compared to TA alone, superiority over surgical treatment has not been proven [10, 11]. Preclinical studies identified potential benefits of combined radiofrequency ablation (RFA) and radiation-based therapies [12–15]. However, the liver has a low tolerability to external beam radiation therapy [16, 17]. TARE provides a selective way of delivering high doses of radiation therapy to a tumor while saving healthy parenchyma [18, 19] and may work synergistically with RFA when the two therapies are combined.

Since RFA induces hyperemia around the ablation zone [20], this reactive viable liver parenchyma corresponds to the volume where residual tumor cells or satellite nodules are most likely to reside, if present [9]. We hypothesized that this hyperemic effect can be used to deliver a high absorbed dose of holmium-166 microspheres (^166^Ho-MS) to the tissue directly bordering the ablated tissue with the aim of decreasing chances of developing local recurrences. Early studies on TARE dosimetry reported on higher response rates in patients who received ≥ 120 Gy of yttrium-90 (^90^Y) monotherapy on their nonresectable HCC, compared to patients who received a lower absorbed dose [21]. The primary objective of this prospective study was to find the treatment volume absorbed dose of ^166^Ho-MS that yields an absorbed dose of ≥ 120 Gy to the hyperemic zone (target volume). Secondary objectives were to investigate safety and efficacy of this adjuvant therapy.

## Materials and methods

### Design

The HORA EST HCC study (NCT03437382) was a multicenter (3 tertiary referral centers for HCC), open-label, non-randomized phase Ib dose-escalation study to the use of adjuvant TARE after RFA in patients with Barcelona Clinic for Liver Cancer (BCLC) early stage HCC (A) lesions of 2–5 cm [2]. The study protocol was approved by the local Medical Ethics Committee and was performed in accordance with good clinical practice and the Declaration of Helsinki. All participants provided written informed consent. The full study protocol has been published earlier, in accordance with good research practice [22].

### Patients

Eligible patients were those with BCLC early stage HCC (A) with a solitary lesion of 2–5 cm or with up to 3 lesions of ≤ 3 cm and at least one lesion > 2 cm, in whom surgical resection was not the treatment of first choice upon decision by the multidisciplinary tumor board. Main inclusion criteria were age of ≥ 18 years old, Child-Pugh (CP) A or B ≤ 7, an Eastern Cooperative Oncology Group (ECOG) performance status of 0 or 1, an estimated TARE treatment volume ≤ 50% of the total liver volume, no prior hemi-hepatectomy or radiation therapy, and a creatinine clearance rate ≥ 30 mL/min. A list of all in- and exclusion criteria can be found in Table 1.

**Table 1:** Inclusion and exclusion criteria

Inclusion Criteria	Exclusion criteria
Informed consent	Tumor location precluding percutaneous RFA
Age > 18 year	Treatment volume > 50% of total liver
Single HCC lesion with diameter of ≥ 2–5cm or up to three lesions with each lesion measuring no more than 3 cm	Vascular tumor invasion or extrahepatic metastasis
HCC diagnosis is based on histology or non-invasive imaging criteria according to EORTC-EASL guidelines	Prior hemi-hepatectomy
Child-Pugh A or B ≤ 7	Severe comorbidity (e.g., cardiovascular disease, diabetes with nephropathy, active infections)
(HCC-unrelated) ECOG performance status ≤ 2	Uncorrectable coagulopathy
Bilirubin < 2 mg/dL	Large arterio-portal venous shunting
ASAT < 5× upper limit of normal	Previous radiotherapy to the liver
ALAT < 5× upper limit of normal	Surgical hepatico-enterostomy
Thrombocytes ≥ 50 × 10^9^/L	Hepatic resection with placement of surgical clips that may cause artifacts on MRI
	Incapability to give informed consent due to mental disorder
	Pregnancy, inadequate anticonception
	Lung shunt fraction > 20%
	Creatinine clearance < 30 mL/min/1.73 m^2^

### Study procedures

**Fig. 1:**
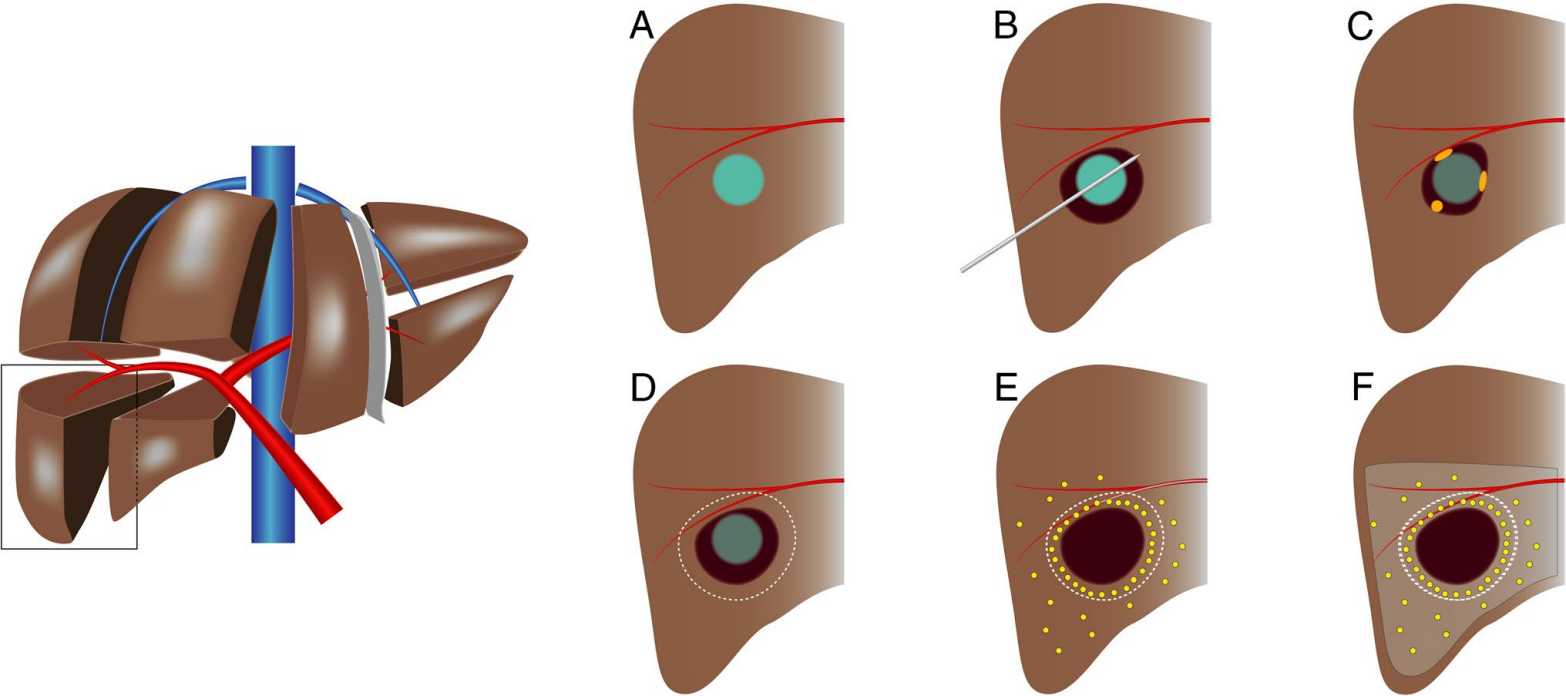
Schematic drawings of the study procedure. **A** HCC lesion of 2–5 cm. **B** Thermal ablation of HCC lesion. **C** Potential sites of local recurrences due to impaired heat propagation, heat-sink effect, or satellite nodules. **D** Target volume for adjuvant TARE. **E** Deposition of ^166^Ho-MS with preferential flow of microspheres to the hyperemic zone surrounding the ablated tissue (i.e., target volume). **F** Liver volume infused with ^166^Ho-MS TARE (i.e., treatment volume) [22].

A schematic overview of the study procedures can be found in Fig. 1. On the first day of treatment, ultrasound or CT guided RFA was performed under general anesthesia using 3 × 3 or 3 × 4 cm exposed tip multi-electrode Cool-tip™ RFA system, electrodes and switching controller (Medtronic Inc, Dublin, Ireland). Immediately after RFA, a contrast enhanced computed tomography (CECT) scan was performed on a 64-slice Aquilion CT-scanner (Canon, Tochigi, Japan) and an additional ablation was acquired in the same session in case residual viable tumor tissue was identified on this scan.

On day 2, an angiography procedure was performed to selectively catheterize the hepatic arteries with vascular supply to the hyperemic tissue using a Progreat 2.4F or 2.7F microcatheter (Terumo corporation, Tokyo, Japan). Catheter position(s) were chosen as selectively as possible and were verified by contrast enhanced cone-beam CT (CBCT). Next, 150 MBq of technetium-99m labeled macroaggregated albumin ([^99m^Tc]Tc-MAA) was injected. The treatment volume was defined as the volume exposed to radiation, based on CBCT [23]. This would include both the hyperemic zone (i.e., target volume) and a limited volume of normal liver parenchyma (i.e., non-target volume). A single photon emission computed tomography (SPECT-CT) scan was acquired directly after the angiography procedure on a Symbia T6 or Symbia Intevo (Siemens Healthineers, Erlangen, Germany) or Discovery 670 Pro (GE Healthcare, Boston, Massachusetts, USA).

On day 5–10 after RFA, TARE with infusion of ^166^Ho-MS QuiremSpheres (Quirem Medical B.V., Deventer, the Netherlands) was performed during a second hospitalization. Prior to ^166^Ho-MS injection, the catheter position was verified using fluoroscopy and CBCT to ensure that spheres would be injected at the identical location as the [^99m^Tc]Tc-MAA. The total activity administered was calculated using the following equation [24]:

$$A_{Ho-166}=Treatment\;volume\;absorbed\;dose\left[Gy\right]\times M_i\lbrack kg\rbrack\times63\lbrack MBq/J\rbrack$$

The treatment volume was segmented from the contrast enhanced CBCT and a tissue density of 1.00 g/mL was used to determine the mass of the treatment volume (*M*_*i*_). One day after TARE (day 6–11), a post-treatment SPECT-CT was acquired for post-treatment dosimetry purposes. These SPECT images were acquired with a medium energy general purpose collimator. A total of 90 projections over a circular 360° orbit were acquired on a 128 × 128 matrix with an overall scanning time of 27 min (18 s per projection). Projections were recorded in the 81 keV (15% width) photopeak window. An additional energy window centered at 118 keV (12% width) was used to correct for bremsstrahlung and higher energy gamma emissions. Planar scintigraphy was used to calculate lung shunting. In addition to this SPECT-scan, MRI was performed before and after TARE to allow MRI-based quantification of ^166^Ho-MS. The MRI-images were acquired on a 1.5T scanner (Ingenia, Philips Healthcare, Best, The Netherlands) and included an MGRE sequence with 10 subsequent echoes (TE1, 1.06 ms; ∆TE, 1.38 ms; TR, 149 ms; flip angle, 33°; in-plane resolution, 2 × 2 mm^2^; slice thickness, 4 mm; FOV, 384 × 384 mm^2^).

### Follow-up

All patients were followed for 12 months which included imaging using CECT or dynamic MRI of the liver and chest at 6 weeks and 3 months after treatment, and every 3 months thereafter. Clinical assessment and biochemical liver function tests were performed at week 2 and simultaneous with all moments of imaging.

### Endpoints

The primary endpoint of this study was to find the treatment volume absorbed dose that resulted in an absorbed dose of ≥ 120 Gy to the target volume in 9/10 patients within a cohort, based on post-treatment SPECT-CT. The target volume was defined as the hyperemic zone encompassing the ablated tissue and generally anticipated to be a 1-cm rim around the ablated tissue. Manual segmentation of the treatment and target volumes in the post-treatment SPECT scan was performed using Xeleris workstation version 4.0 (GE Healthcare, Boston, Massachusetts, USA). The non-target volume dose was defined as the treatment volume subtracted by the target volume. Post-treatment MRI dosimetry was performed using Q-Suite 2.0 software (Quirem Medical B.V. Deventer, The Netherlands).

In the first cohort, a dose of 60 Gy was administered to the treatment volume. If a second patient within a cohort failed to reach an absorbed dose of ≥ 120 Gy to the target volume, the dose was escalated to 90 Gy to the treatment volume in subsequent patients (cohort 2) and could ultimately be escalated to 120 Gy (cohort 3). The design of this study was based on the assumption that microspheres would preferentially flow to the hyperemic zone around the ablation zone (i.e., target volume) rather than to the normal parenchyma (i.e., non-target volume) within the treatment volume. If the ratio of microsphere accumulation in the target volume versus normal non-target volume would be high, a low amount of radioactivity to the treatment volume (cohort 1) would be sufficient to reach an absorbed dose of ≥ 120 Gy to the target volume. If there would be an even distribution of microspheres between the target volume and non-target volume a treatment volume absorbed dose of 120 Gy (cohort 3) would be needed to meet the study endpoint. Per cohort at least 2 patients were treated and no further dose escalation was performed when the final endpoint was met of an absorbed dose of ≥ 120 Gy to the target volume in 9/10 patients. The sample size of this study was thus determined to be a minimum of 10 and a maximum of 30 patients.

Secondary endpoints included toxicity, local tumor recurrence rates, progression-free survival (PFS), and overall survival (OS) at 6 months and at 1 year. Adverse events were categorized according to Common Terminology Criteria for Adverse Events (CTCAE) 4.0 [25]. Local recurrences were defined as appearance at follow-up of foci of untreated disease in tumors that were previously considered to be completely ablated, in concordance with the CIRSE Standards of practice guideline [26].

MRI-based quantification of ^166^Ho-MS was investigated as an exploratory endpoint.

### Statistical analysis

Descriptive statistics and outcomes were calculated by medians and interquartile ranges (IQR) for continuous variables and frequencies and percentages per category for categorical variables. Local recurrence free survival, PFS, and OS rates at 6-month and 12-month follow-up were calculated. Patients that underwent liver transplantation were censored in the survival statistics. Statistical analyses were performed using RStudio 1.4.1106.

## Results

### Patients

**Fig. 2:**
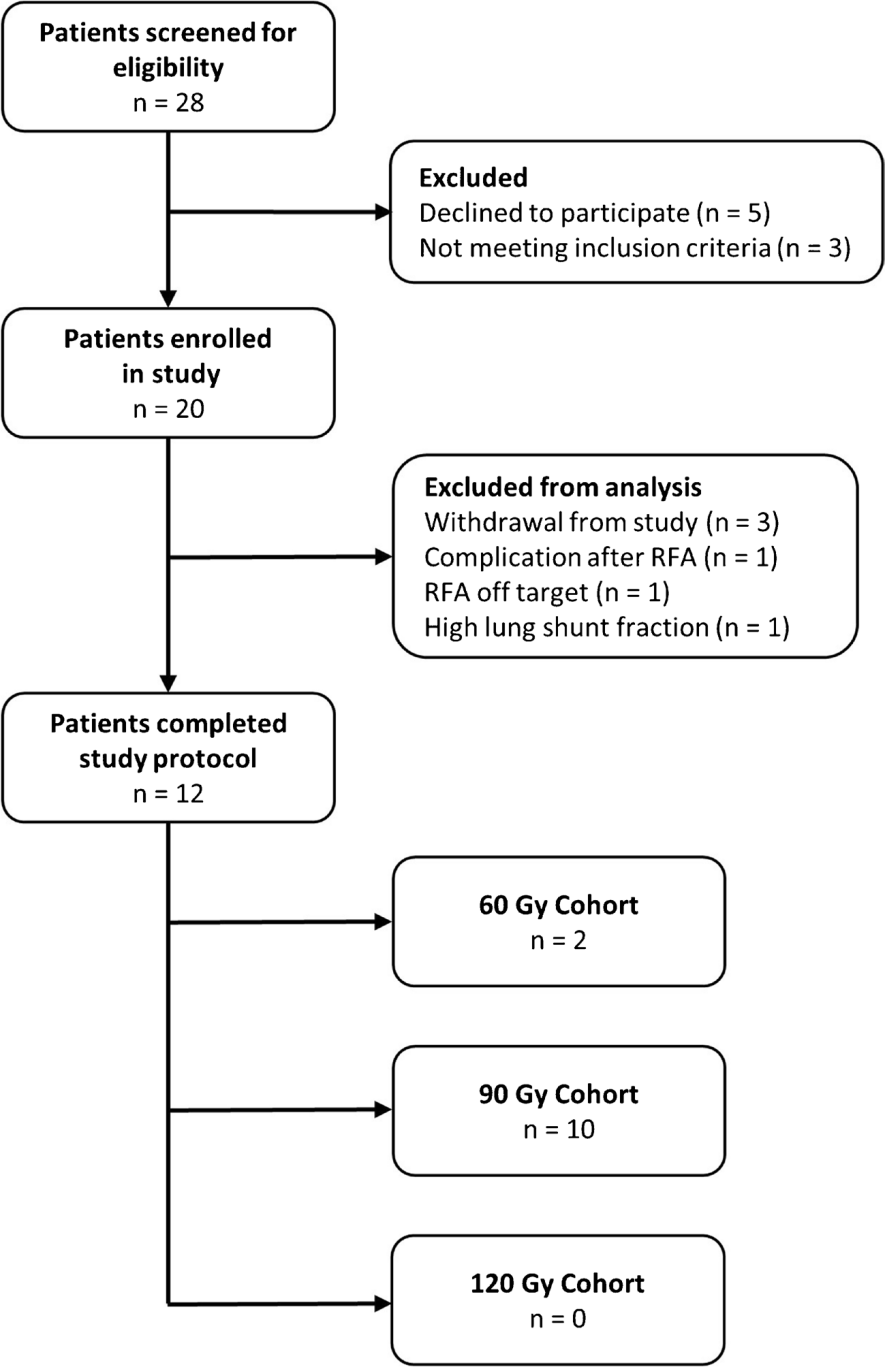
Flowchart of the study population.

Informed consent was obtained from 20 patients between April 2018 and March 2021. Twelve of these patients completed the treatment regimen, as can be seen in Fig. 2. Reasons for exclusion were withdrawal from the study (*n* = 3), progression beyond BCLC early stage HCC in the time between inclusion and treatment (*n* = 1), CTCAE grade 3 complication after RFA (*n* = 1), RFA off target (*n* = 1), high lung shunt fraction (*n* = 1), and incomplete administration of ^166^Ho-MS (*n* = 1). Baseline characteristics of all 12 treated patients are shown in Table 2. The population consisted of more males (*n* = 10) than females (*n* = 2) and most patients had underlying Child-Pugh A liver cirrhosis (*n* = 10).

**Table 2:** Patient characteristics of analyzed patients

		*n*	
Total		12	
Age	*Median [IQR]*	66.5	[64.3–71.7]
Sex	Male	10	83%
	Female	2	17%
Liver parenchyma status	Child-Pugh A cirrhosis	10	83%
	Fibrosis	2	17%
Etiology of cirrhosis	Hepatitis B	4	40%
	Alcohol induced	6	60%
BCLC stage	Early	12	100%
Prior HCC treatment	None	11	
	TA	1	
Number of study lesions*	1	11	92%
	2	1	8%
Tumor location (Couinaud segments)	Segment 3	1	
	Segment 4	2	
	Segment 5	1	
	Segment 6	2	
	Segment 7	6	
	Segment 8	1	
Size (mm) of study lesions*	*Median [IQR]*	27	[21–40]

^*^3 lesions in 2 patients were treated with TA only in the same treatment session. All three lesions were < 15 mm and therefore not eligible for TARE after TA. *HCC* hepatocellular carcinoma, *BCLC* Barcelona Clinic for Liver Cancer, *TA* thermal ablation, *TACE* trans-arterial chemoembolization

### Treatment

**Fig. 3:**
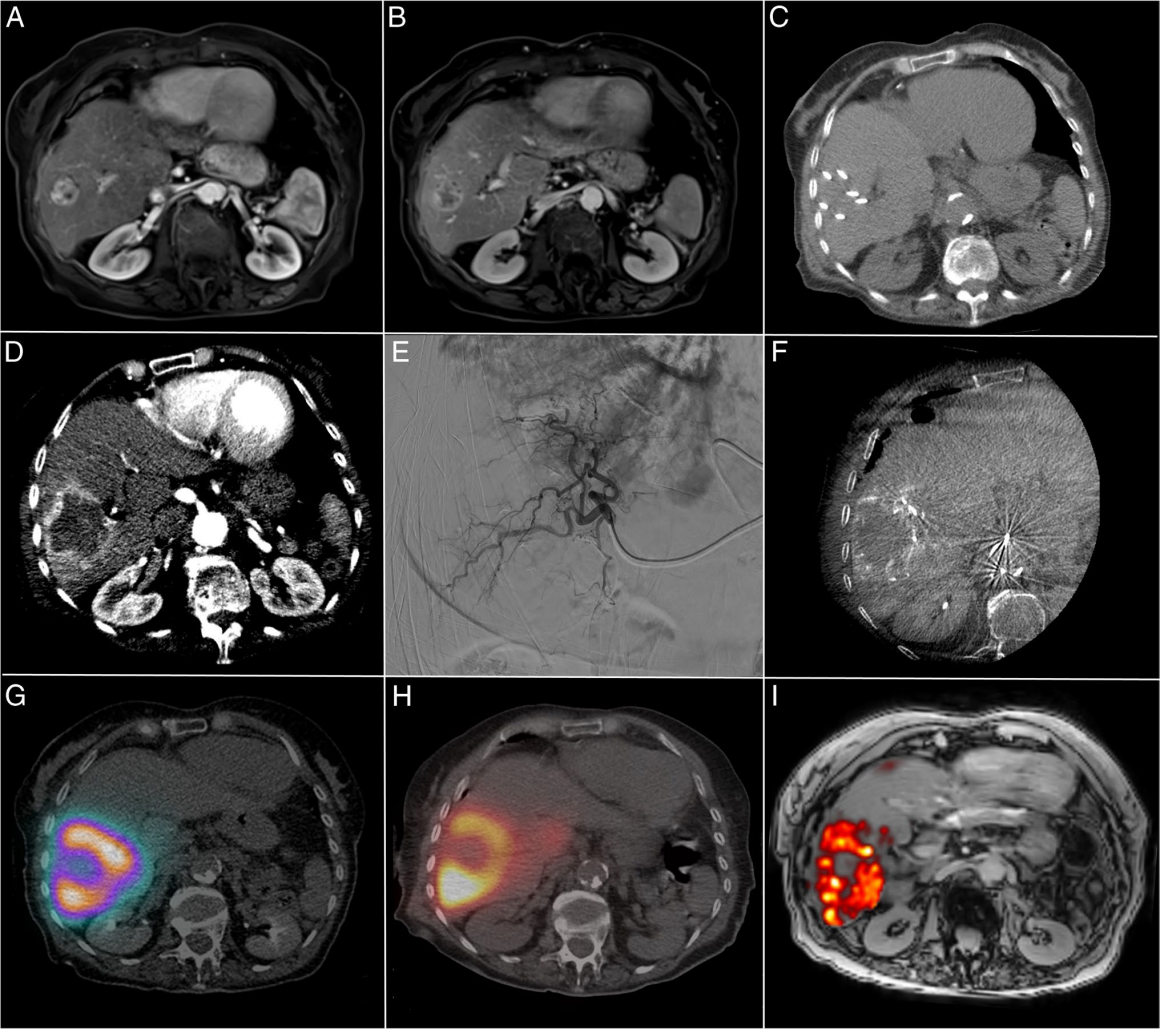
HORA EST HCC treatment sequence: **A** Arterial scan phase of diagnostic MRI showing a hypervascular HCC lesion of 31 mm in the liver. **B** Portal venous scan phase of MRI showing central wash-out in the HCC lesion. **C** Intraprocedural CT after placement of six cooled-tip RFA needles with 3-cm exposed tip. **D** Intraprocedural contrast enhanced CT scan in arterial phase showing hyperemia around the ablation zone on post-ablation CECT. **E** Super-selective catheterization of hepatic arteries with vascular supply to the target volume. **F** CBCT of the treatment volume with an identical catheter position as in **E**. **G** SPECT-CT of [^99m^Tc]Tc-MAA dose distribution used for dose planning. **H** SPECT-CT of ^166^Ho-MS distribution. **I** MRI-based dosimetry of ^166^Ho-MS distribution.

A patient case example is given in Fig. 3. Treatment characteristics can be found in Table 3. All ablations were performed with a multiprobe approach. Three out of 16 lesions in two out of 12 patients were treated with RFA only as the tumor diameter was < 2 cm. In those patients, only the larger lesion(s) (> 2 cm) were treated with adjuvant TARE after RFA. Most ^166^Ho-MS infusions were performed (sub-)segmental or bi-segmental, and one infusion was performed lobar. The median treatment volume was 360 mL (IQR, [270–394]), and the median administered activity of ^166^Ho was 1.79 GBq (IQR, [1.45–2.23]).

**Table 3:** Treatment characteristics

		*n*	
RFA probes used	Multiprobe 3 × 3 cm	5	
	Multiprobe 3 × 4 cm	3	
	Multiprobe 6 × 3 cm	2	
	Multiprobe 6 × 4 cm	2	
Modality used for needle placement	CT	2	
	Ultrasound	10	
Angiography: catheter position	(sub-)segmental	2	
	bi-segmental	9	
	lobar	1	
Treatment volume (mL)	Median [IQR]	360	[270–394]
Target volume (mL)	Median [IQR]	88	[69–128]
Lung shunt fraction (%)	Median [IQR]	4.6	[2.2–6.55]
Dose to treatment volume	60 Gy	2	
	90 Gy	10	
	120 Gy	0	
Administered activity of ^166^Ho (GBq)	Median [IQR]	1.79	[1.45–2.23]

*RFA* radiofrequency ablation, *CT* computed tomography, ^*166*^*Ho* holmium-166, *mL* milliliter, *GBq* Giga-becquerel, *Gy* gray

### Primary endpoint

**Fig. 4:**
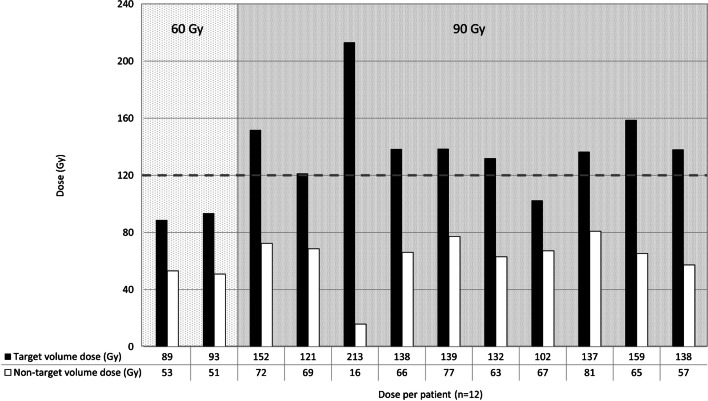
Dose distribution per patient within treatment volume, based on SPECT imaging. The bars in black represent the mean absorbed dose on the target volume directly surrounding the ablation volume per patient. The cutoff point of an absorbed target volume dose of $\ge$ 120 Gy is indicated by the horizontal dashed line. The bars in white show the absorbed dose to the non-target volume within the treatment volume. The first two patients were treated with 60 Gy to the treatment volume, whereas the other patients were treated with 90 Gy to the treatment volume. The median ratio of target volume dose vs non-target volume dose was 1.97 (IQR, [1.75–2.17]).

The first two patients were treated with a dose of 60 Gy on the treatment volume. Figure 4 shows the dose distribution per patient. Although a preferential dose accumulation in the target volume was found in the first two patients, the absorbed target volume doses were 89 Gy and 93 Gy, respectively. As the endpoint of ≥ 120 Gy to the target volume was not met, the dose was escalated to 90 Gy to the treatment volume. In 9/10 patients in the 90 Gy cohort, a mean target volume dose of ≥ 120 Gy was met. In this cohort the median absorbed target volume dose was 138 Gy (IQR, [127–145]), and the median absorbed non-target volume dose was 67 Gy (IQR, [54–75]), as can be seen in Fig. 4. As the primary endpoint was met, the inclusion was closed and the recommended treatment volume absorbed dose was set at 90 Gy.

### Toxicity

One patient was readmitted to the hospital on the third day after radioembolization because of fever. Ultrasound and CECT demonstrated abscess formation within the ablated tissue that was treated with percutaneous drainage (CTCAE 4.0 grade 3 infection). Other reported adverse events were grade 1–2 nausea (*n* = 3) and grade 1 fatigue (*n* = 4).

### Efficacy

**Fig. 5:**
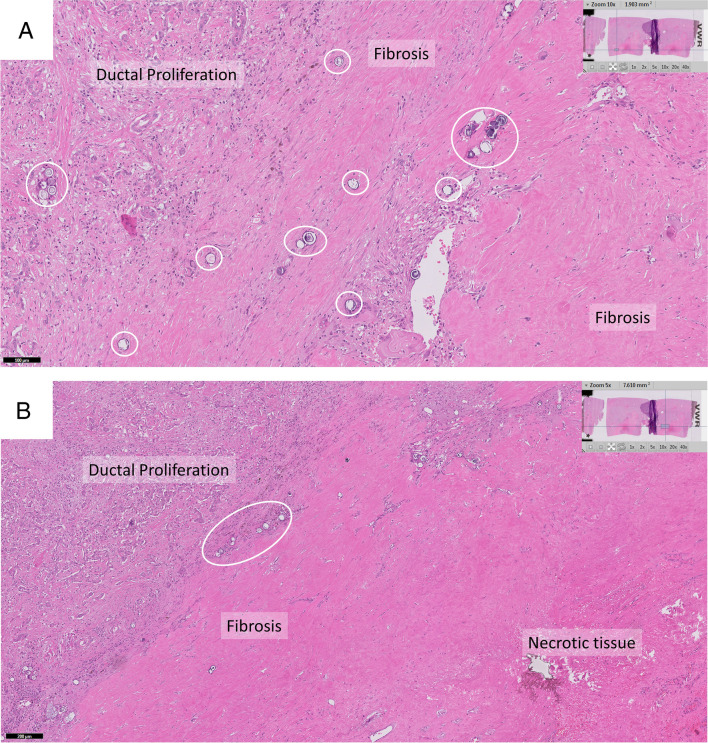
Histology of explanted liver treated with radiofrequency ablation and adjuvant ^166^Ho TARE. Digitalized histology using Ultra Fast Scanner (Philips Healthcare, Best, The Netherlands) with a magnitude of 40×. **A** Zoom 10×. Transition from liver tissue with ductal proliferation to fibrosis with marked depositions of ^166^Ho-MS. **B** Zoom 5×. Overview of transition from ductal proliferation to necrotic tissue with marked ^166^Ho-MS.

Two patients underwent liver transplantation at 7.5 and 8.0 months after treatment. They were both local recurrence free before liver transplantation. All other ten patients were also free of local recurrences within 12 months after treatment. Three patients developed new HCC lesions elsewhere in the liver, at 4.6, 5.5, and 5.6 months. Therefore, PFS was 75% at 6 months and 75% at 1 year. Two patients died, one as a result of decompensated liver cirrhosis and one following bacterial sepsis after liver transplantation. This resulted in an OS of 92% at 6 months and 83% at 1 year. Figure 5 shows an example of histological confirmation of ^166^Ho-MS accumulation surrounding the fibrotic and central necrotic tissue.

## Discussion

In this multi-center, single arm study we prospectively evaluated the feasibility of adjuvant TARE after RFA in BCLC early stage HCC 2–5 cm. The results show that an absorbed dose of > 120 Gy of ^166^Ho-MS on the target volume around the ablation zone could be reached at an administered dose of 90 Gy to the treatment volume. The median target volume dose was about twice as high as the median dose to the non-target parenchyma, confirming our hypothesis that hyperemia induced by RFA can be utilized to deposit ^166^Ho-MS in a peripheral zone surrounding the ablation volume. The safety profile of the combined treatment was in concordance with the safety of RFA or TARE mono-therapy, or combined RFA and TACE. Only one CTCAE grade 4 complication occurred in 12 patients (8.3%) and no grade 5 complications were observed [27–29]. Within 1 year after treatment, no local recurrences developed, three patients developed recurrent HCC elsewhere in the liver and two patients died. Treatment efficacy and safety profile should be further validated in a larger cohort.

Many patients with larger HCC lesions are not eligible to surgical resection due to comorbidities, cirrhosis with portal hypertension, or insufficient future liver remnant volume. TA is an alternative treatment, but a large diameter is an important risk factor of local recurrence [6, 8]. In the continuous search towards better treatment outcomes and extended bridging to liver transplantation, several treatment combinations of TA with other locoregional or systemic therapies have been investigated. The STORM trial investigated adjuvant sorafenib after surgery or TA, but failed to prove benefit in terms of time to progression free and overall survival [30]. Another widely studied combined treatment regimen is TA with (neo)adjuvant TACE. Several trials in Asian populations have indicated superiority of combined TA and TACE over TA alone [31, 32], but the combination therapy has not been adopted in the EASL, AASL, or BCLC guidelines [1, 2, 7]. The different studies have methodological limitations and there is a considerable variation between the trials in technique and treatment sequence [33–35]. Furthermore, superiority of the combination therapy over surgical resection has not been proven [11, 33]. To our knowledge this is the first study to combine TA with TARE.

Technical advancements have led to the adoption of TA as the preferred treatment of HCC < 2 cm a decade ago [2, 36]. Similarly, recent advancements in patient selection and optimized patient-tailored dosing have resulted in a place for TARE in the recent BCLC update [2]. The LEGACY and RASER studies reported promising results of radiation segmentectomy in patients with (very) early stage HCC patients with a mean lesion diameter of 2.7 cm and median lesion diameter of 2.1 cm, respectively [37, 38]. These results indicate high local control rates to be achievable using radiation segmentectomy, although results were not superior to those that may be achieved with TA. Further prospective validation is needed in larger trials and in patients with larger lesions. Ultimately, the role of TARE in HCC is to be further clarified for different indications.

In this trial, only RFA was used as ablation modality. In this way, the treatment regimen was kept as homogeneous as possible. Moreover, preclinical work combining TA with radiation-based therapies was only performed with RFA [12–15]. However, over the last years, the use of microwave ablation (MWA) has increased. MWA may have some technological advancements over RFA, but similar outcomes have been found [39]. As hyperemia around the ablation zone is seen after MWA similarly to RFA, it is expected that a similar ^166^Ho-MS dose distribution can be achieved when TARE is performed following MWA [40–42].

^166^Ho-MS were used for radioembolization in this study rather than ^90^Y TARE. ^166^Ho has advantages in terms of imaging as it emits direct gamma radiation at 81 keV. Moreover, the paramagnetic property of ^166^Ho allows for MRI-based post TARE dosimetry [24, 43]. The study endpoint was determined using SPECT-based dosimetry, and MRI-based quantification of ^166^Ho-MS was used as an exploratory endpoint. Unfortunately, reliable quantitative MRI-based dosimetry was unfeasible in many patients as a result of breathing and movement artifacts. MRI scans were obtained shortly after the RFA and TARE procedures, and many patients experienced discomfort and as a result had difficulty lying still and maintaining breath holds. [^99m^Tc]Tc-MAA was used for the scout procedure. Despite the potential benefits of ^166^Ho scout dose in terms of intrahepatic treatment dose distribution mimicking, ^166^Ho scout dose was not yet available by the time of study design [44]. In the current study, however, standard volume-based dosimetry was used based on CBCT, so this would not have affected dose planning

The current study has several limitations. First, the sample size is small and therefore no definite conclusions can be drawn on the efficacy. Nevertheless, the absence of local recurrences in all study patients within 1 year after treatment suggests that the efficacy of the combination therapy is high. Second, despite of meeting the primary end point at an administered dose of 90 Gy, a substantial variety in ratio of target volume dose versus non-target volume dose between individual patients was observed. This ratio depends on various factors, such as degree of hyperemia, catheter position, occurrence of vascular stasis during injection, and the ratio target volume versus treatment volume. In the 90 Gy cohort, an absorbed target volume dose of ≥ 120 Gy was not reached in one patient. As a result of a very selective catheter position in this patient, the target volume constituted > 50% of the treatment volume. In patients where the ratio between target volume and treatment volume ratio is very high, an administered dose higher than 90 Gy to the treatment volume may be required. Clearly, in the theoretical case that the target volume constitutes 100% of the treatment volume, TARE with a dose of 90 Gy would not be sufficient. For future studies, a volume-dependent administration dose planning could help to individualize treatment planning. Another limitation of this study is the complexity of the treatment regimen. For patients this meant undergoing a second treatment, including four additional imaging examinations (2× MRI and 2× SPECT/CT) and an additional hospitalization. This was considered burdensome by some patients, and therefore a reason not to participate in this trial.

Since the initial plans of this study originate from 2017, less was known on (^166^Ho) TARE dosimetry, and the 120 Gy cutoff was mainly chosen based on initial ^90^Y research [21]. Treatment volume absorbed doses of the several cohorts were based on a phase I ^166^Ho-MS dose escalation study (HEPAR trial), in which a whole liver dose of 60 Gy was considered safe [45]. Recently, the first efficacy evidence for ^166^Ho-MS in HCC was demonstrated in the HEPAR Primary study [28]. At a treatment volume absorbed dose of 50 Gy in an average of 54% of the total liver volume, partial or complete responses were seen in patients receiving an average absorbed dose of 210 Gy on their lesions versus 116 Gy in patients with progressive disease [28]. Since hyperemic tissue surrounding the ablation zone is targeted in our study rather than (large) lesions, these tumor dose values cannot be directly compared to the 138 Gy absorbed target volume dose found in our study. Nevertheless, taking into account the recent advancements of safe radiation segmentectomy procedures, and the fact that a tumor absorbed dose of 210 Gy did show a higher level of tissue necrotization when compared to an absorbed dose of 116 Gy in the HEPAR primary study, investigating higher dosing of ^166^Ho-MS as adjuvant treatment after thermal ablation seems to be justified. Especially when a small treatment volume is treated that mainly consists of the target volume, a higher dose than in the current trail should be chosen. In light of recent segmentectomy studies [37, 38, 46], recommendations with ^90^Y [47], and the HEPAR primary trial [28], the treatment volume absorbed dose may be as high as about 200 Gy. For patients with a larger treatment volume (for example due to multiple ablations or a more centrally located tumor), a treatment volume absorbed dose of 90 Gy remains recommended to limit the absorbed radiation dose to the liver parenchyma. Our study provides insight in the biodistribution of ^166^Ho-MS after TA with an average target volume vs non-target volume ratio of 2:1. This may help to determine the optimal dose in each individual patient, while taking into account the risk of radiation induced liver disease in patients with a larger treatment volume.

The median tumor diameter in this study was 2.7 cm. Patients with a tumor diameter of ≥ 2 cm were eligible for inclusion in this dose finding study. It may be questionable whether adjuvant TARE will be cost-effective in patients with a tumor < 3 cm. Future studies investigating effectivity of thermal ablation with adjuvant TARE are more likely to be positive when larger tumors are recruited.

Moreover, in a future study, the feasibility of combined TA and TARE in a single procedure could be explored. Owing to the low dose of ^166^Ho-MS used in this treatment regimen, the chance of introducing a substantial radiation dose to the lung parenchyma is extremely low. Moreover, as a result of super-selective catheterization and the use of CBCT prior to infusion of ^166^Ho-MS, the chance of other extrahepatic deposition is small as well. Especially since combined Angio-CT systems are increasingly being used, the combined treatment could be performed in a single session with high precision [48]. The current proposed treatment protocol is promising for the locoregional treatment of HCC lesions 2–5 cm that are at higher risk of local recurrences. Further research into subtypes of HCC or identification of satellite nodules may contribute to identifying patients who potentially benefit most of the combined treatment regimen.

## Conclusion

Selective radioembolization with ^166^Ho-MS can be used safely as an adjuvant treatment in early stage HCC 2–5 cm. Hyperemia induced by TA can be utilized to deliver a high radiation dose to the target volume while limiting the dose to the normal liver parenchyma. A treatment volume absorbed dose of 90 Gy is safe and sufficient to deliver a tumoricidal absorbed radiation dose of at least 120 Gy to the target volume.

## Data Availability

The datasets generated during and/or analyzed during the current study are available from the corresponding author on reasonable request.
